# Utilizing Nutritional and Polyphenolic Compounds in Underutilized Plant Seeds for Health Application

**DOI:** 10.3390/molecules27206813

**Published:** 2022-10-12

**Authors:** Nur Syamimi Zaini, Roselina Karim, Ahmad Faizal Abdull Razis, Norhasnida Zawawi

**Affiliations:** 1 Functional Carbohydrate Research Laboratory, Faculty of Food Science and Technology, Universiti Putra Malaysia, Serdang 43400, Selangor, Malaysia; 2 Department of Food Technology, Faculty of Food Science and Technology, Universiti Putra Malaysia, Serdang 43400, Selangor, Malaysia; 3 Natural Medicines and Products Research Laboratory, Institute of Bioscience, Universiti Putra Malaysia, Serdang 43400, Selangor, Malaysia; 4 Laboratory of Halal Science Research, Halal Products Research Institute, Universiti Putra Malaysia, Serdang 43400, Selangor, Malaysia

**Keywords:** fibre crops, underutilized food sources, nutritional values, bioactive compounds, sustainability

## Abstract

Plants represent a significant part of the human diet. Humans have utilized every part of plants for survival, and seeds are no exception. Seeds offer high protein, unsaturated fats, fibre, essential vitamins, and minerals for various food applications. They are also a promising reservoir of bioactive compounds, where various phytochemicals, such as polyphenolic compounds, capable of maintaining and improving well-being, are present in abundant quantities. Plants from Malvaceae and Cannabaceae families are known for their fibre-rich stems that benefit humankind by serving numerous purposes. For many centuries they have been exploited extensively for various commercial and industrial uses. Their seeds, which are often regarded as a by-product of fibre processing, have been scientifically discovered to have an essential role in combating hypercholesterolemia, diabetes, cancer, and oxidative stress. Maximizing the use of these agricultural wastes can be a promising approach to creating a more sustainable world, in accordance with the concept of Sustainable Development Goals (SDGs).

## 1. Introduction

The global population is growing fast, and so is the demand for food [1]. Food production needs to be heightened through agricultural practices, and utilization of their by-products is essential to achieving food security [2]. In accordance with the Food and Agriculture Organizations [3] of the United Nations, a sustainable food system can ensure food security without compromising the future generations’ food security by minimizing negative impacts on the economy, society, and the environment. The Sustainable Development Goals (SDGs), which aim to eradicate hunger and malnutrition of all forms (Goal 2: Zero Hunger), on top of reducing food waste (Goal 12: Responsible Consumption and Production), should be achieved by 2030 [4]. These goals can be achieved by reducing energy usage in food production and food waste. Livestock rearing involves releasing greenhouse gases (GHG) and land use, which puts ecosystems at risk of acidification and eutrophication [5,6]. Increase in production and consumption of plant-based foods and added-value food products made from agricultural wastes have been shown to make a significant contribution towards reducing the aforementioned environmental impacts [7,8,9,10].

Further, the global health burden has shifted from infectious or communicable diseases to non-communicable diseases (NCDs) over the last century. According to the World Health Organization (WHO), some NCDs, such as ischemic heart disease, stroke, and diabetes mellitus, have entered the top ten causes of death worldwide [11]. Unfortunately, a new lethal virus, known as a Coronavirus (COVID-19), has been circulating since the end of 2019 and has robbed the lives of millions of people, particularly those with underlying comorbidities. They were immunocompromised due to unsatisfactory heart health and blood sugar control, making them very vulnerable to infection and giving them a smaller chance of recovery [12]. People are increasingly more concerned about their health due to these factors. Therefore, lifestyle adjustments such as acquiring the correct amount of carbohydrates and protein, lowering saturated and trans-fat intake, and eating plenty of fruits and vegetables may be beneficial in combating both the NCD epidemic and the COVID-19 pandemic.

Plants are unique living beings due to the fact that they have different parts, including leaves, flowers, stems, roots, and seeds, which are very useful to humankind as a source of a wide range of nutrients. Although they are principally small in size, seeds serve many vital functions for the plants themselves (e.g., to store nutrients) and animals and humans (e.g., as a food source). At present, many plants that are noteworthy for their fruits and fibre are processed and their other parts, including seeds, are left as by-products or waste materials [13]. As a matter of fact, seeds are considered as part of food loss and waste (FLW), in accordance with FAO [4]. According to international statistics, it is estimated that an annual production of FLW is 1.3 billion tons worldwide, however, these should be properly managed to reduce the environmental footprint and improve global health. Seeds have several layers that possess protective properties to allow them to survive in extreme conditions and be protected from predators. The outer layer is abundant with micronutrients such as vitamins and minerals and a surplus of biologically active chemical compounds that produce therapeutic activities. This layer also protects the endosperm inside, where the most valuable nutrients (e.g., protein, carbohydrate, and fat) are concentrated [14].

Other than essential nutrients, plant seeds hold many naturally occurring bioactive components, namely polyphenols, flavonoids, and phytosterols, that play essential roles in preventing and treating diseases. As NCDs that arise from oxidative stress and inflammation are emerging and continuously burdening individuals and societies, healthy dietary intake from plant sources with better fatty acid composition (lower in saturated fats, higher in unsaturated fats) and which are richer in antioxidants as compared to animal sources can assist in combating many health problems [5,15,16]. Hypertension, hypercholesterolemia, diabetes, and diet-related cancers result from oxidative stress and inflammation induced by high-fat and low antioxidant diets [5,15,16]. Consequently, the mortality rate can also be reduced, following Goal 3 of the SDGs, ‘to ensure healthy lives and promote well-being for all ages’.

The amount of research conducted on the use of agricultural waste, such as the seeds, to create a more sustainable world is increasing. However, the literature on the valorisation of the bioactive components is relatively limited, particularly regarding polyphenolic compounds from seeds. An example is oilseeds, which are seeds that are cultivated primarily to provide edible oils. These seeds are often discarded in landfills, where they decompose and emit greenhouse gases, despite being an attractive source of polyphenols (in addition to protein and fibre) with their excellent bioactivities [17]. Other underutilized seeds such as seeds from the plant okra (*Abelmoschus esculentus* L.), cotton (*Gossypium* spp.), hemp (*Cannabis sativa* L.), and kenaf (*Hibiscus cannabinus* L.) share comparatively similar specialties and advantages in containing nutrients beyond the essentials. They can be exploited as an energy source and building blocks for growth obtained from available macronutrients, and for prevention of non-communicable diseases through other acquired nutrients such as dietary fibre, vitamins, minerals, and a surfeit of phytochemicals. Hence, these plant seeds and derived food products can be termed ‘functional foods’, an established and continuously growing trend in the food and agriculture industries [18]. Although seeds of plants from Malvaceae and Cannabinaceae families have been widely researched throughout the last few decades, their information is discussed individually in the existing body of knowledge. Thus, this review article will summarize their cultivation history, characteristics, application in foods, nutritional benefits with specific focus on their polyphenolic contents, and actions against non-communicable diseases.

## 2. Development of Sustainable Functional Ingredients and Functional Foods from Plant By-Products

By-products generated from various stages of agricultural practices such as fruit peels, fruit pomace, seeds, and cereal brans are rich in bioactive phytonutrients that can alternatively be recovered for many applications (e.g., as antioxidant sources in food supplements and cosmetic products) rather than being discarded and polluting the environment [19]. Polyphenols are among the many sources of phytonutrients in the plant kingdom that can be extracted through solvent extraction (e.g., water, ethanol, and supercritical fluid) or solvent-free extraction (e.g., microwave-assisted and ultrasonic-assisted) modes [20,21]. Currently, there is a rising trend in using natural antioxidants as a replacement to synthetic antioxidants such as butylated hydroxyanisole (BHA), butylated hydroxytoluene (BHT), and tert-butyl hydroquinone (TBHQ) in food preservation and packaging [22]. Although synthetic antioxidants are relatively cheaper and more stable than their natural counterparts, the safety of these chemicals is a matter of great concern [23]. The use of synthetic antioxidants has been associated with a broad spectrum of environmental pollution (e.g., water contamination and poor air quality) and health hazards (e.g., allergies, oxidative stress, and DNA damage) following immoderate exposure [24,25].

Currently, there are various eco-friendly methods that have been developed and can be employed for the efficient extraction of antioxidants from natural matrices such as plants, for example using deep eutectic solvents (DES) in addition to microwave-assisted (MAE), ultrasound-assisted (UAE), and supercritical fluid extractions (SFE). The microwave-assisted extraction approach is beneficial in increasing extraction yield and antioxidant activity of extracted phenolic compounds. It is also more efficient (i.e., shorter extraction time and lower temperature) than conventional extraction methods. Weremfo et al. [26] compared MAE (using 58% ethanol for 5 min at 400 W) to conventional extraction (employing 56% ethanol for 23 min at 63 °C). Microwave-assisted extraction demonstrated increased total phenolic content (by 58.8%) and antioxidant activities according to 1-diphenyl-2-picrylhydrazyl (DPPH) and ferric reducing antioxidant power (FRAP) assays (by 21–25%) from avocado seeds. Moreover, phenolic compounds such as rutin, catechin, and syringic acid were also extracted from avocado seeds.

The supercritical fluid extraction (SFE) method is efficient and affordable; even just a tiny amount of phenolic compounds in plants can be retrieved. Furthermore, this process requires little or no solvent, and heat-sensitive bioactive constituents will be sustained throughout the extraction process. Buszewski et al. [27] discovered many phenolic compounds from *Lupinus luteus* seed extracts by utilizing carbon dioxide under supercritical conditions (Sc-CO_2_). The extracts showed substantial antioxidant and antiradical properties, particularly rich in apigenin and fisetin. Furthermore, they were also non-cytotoxic and had antimicrobial characteristics.

In a recent study, Wu et al. [28] extracted antioxidants from *Polygonum aviculare* leaves using choline chloride and levulinic acid in a deep eutectic solvents-based ultrasonic-assisted extraction (ChCl-Lev-based UAE) method. As opposed to extractions by maceration, Soxhlet apparatus, and microwave, ChCl-Lev-based UAE extracted phenolics such as gallic acid, 5-caffeoylquinic acid, and 3-chlorogenic acid more efficiently. Deep eutectic solvents (DES) are more stable, less volatile, less toxic, and more biodegradable than conventional solvents such as methanol, acetone, and chloroform.

Contrasted with synthetic antioxidants, recovering polyphenols from natural sources does not involve using hazardous chemicals and emission of contaminants (e.g., greenhouse gases) into the atmosphere [24]. High quantities of polar polyphenolic compounds can still be efficiently extracted from plant matrices by simply using water, the ‘greenest’ and cheapest solvent of all [29]. Simple alcohols such as ethanol are also low in toxicity, biodegradable, low cost, and are thus an environmentally preferrable solvent [30]. The advantages concerning health and safety of supercritical fluids such as water and carbon dioxide are prominent, as described by Knez et al. [31]. They have also been affirmed as ‘generally recognized as safe’ (GRAS). Many novel methods applying solvent-free extractions have been used widely in agri-food and nutraceutical industries. The efforts to preserve natural antioxidants are seen to accelerate our progress in meeting Goal 13 of the SDGs on climate action, that is, to take action to combat climate change and its impacts. Therefore, valorisation of underexploited plant sources for their value-added polyphenols could catalyse the global agenda to achieve not only Goal 13 but also the rest of the SDGs.

Seeds are not just essential for plants to produce the next generation but also contain commercial values because of their high nutritional values and functional properties. These properties rendered the usage of seeds into a variety of valuable products such as food ingredients for humans and animal feeds in the forms of seed oil, seed meal, seed milk, and more [32,33]. For that reason, they have become a subject of interest in food science studies as part of the attempts to manage agricultural by-products while meeting nutritional needs and food demands from our growing global population [34]. Wide-ranging studies have been conducted to investigate the beneficial health effects of plant seeds. For example, referring to Ros and Hu [14], higher consumption of plant seeds as well as grains, nuts, legumes, cocoa, and coffee beans can reduce the risk of chronic diet-related ailments such as cardiovascular diseases (CVD) and type 2 diabetes mellitus (T2DM). An abundance of plant seeds has been discovered to participate in chronic disease prevention, attributed to their antitumor, antidiabetic, antioxidant, and antiproliferative effects [35].

## 3. Seeds: An Excellent Source of Polyphenolic Compounds

Polyphenolic compounds or polyphenols are naturally occurring elements with phenol units found exclusively and abundantly in plant-based foods (fruits, vegetables, cereals, grains, legumes, oilseeds, coffee, and tea), alongside their essential nutrients. They are secondary metabolites of plants with antioxidant and antiviral capabilities, produced to protect themselves against pathogens, support cell growth and division processes, and stimulate photosynthesis [36]. Polyphenols are among other secondary metabolites (i.e., terpenoids and isoprenoids, alkaloids, and glucosinolates) biosynthesized in plants by the seven-step shikimic pathway usually occurring in the chloroplast [37]. They are naturally present in specialized cells that are not required for primary photosynthesis or cellular respiration metabolism but are hypothesized to be imperative for plant survival in the environment. Secondary metabolites in plants defend against external factors such as ultra-violet radiation, pathogens, predators, oxidative stress, and extreme climatic conditions. Plants, unlike animals, cannot escape their biotic and abiotic stimuli because their root system attaches them to the soil. Plant phenolics can also be classified into two types: preformed phenolics or induced phenolics. Preformed phenolics are synthesized during normal plant tissue development, whereas plants synthesize induced phenolics in response to the aforementioned external stressors [38].

In addition to performing roles in physiological processes in plants, polyphenols also contribute to their sensorial characteristics, particularly colour, flavour, aroma, and astringency. For instance, flavonoid-type polyphenols are the agents that impart a specific colour to different parts of plants, making them widely known as natural pigments [39]. Flavonoids have also been reported to provide positive health effects, for example in an in vivo study by Zhu et al. [40], it was demonstrated that flavonoids obtained from guava leaves (guaijaverin and avicularin) had antihyperglycemic, antihypercholesterolemic, and hepatic protective effects in diabetic mice. These beneficial effects were linked with inhibition of dipeptidyl peptidase IV by guaijaverin and reduction of glucose uptake (through glucose transporter type 4) by avicularin.

**Figure 1:**
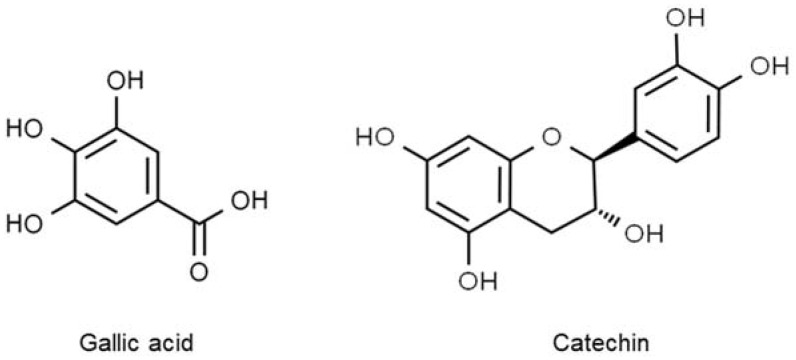
The structures of the most common polyphenols. Gallic acid, C_7_H_6_O_5_ (**left**), belongs to the phenolic acid class, whereas catechin, C_15_H_14_O_6_ (**right**), is part of the flavonoid class.

Polyphenols are broadly diverse in structure. They are all characterized by multiple phenol rings of at least three to five units and several hydroxyl groups. Several examples of polyphenols are gallic acid, isoflavones, tannins, and anthocyanins, which are commonly present in most plants (Figure 1). They have been extensively exploited for many purposes, such as in the manufacture of dyes, ink, and supplements. The number of phenol rings and the type of linkages between the rings determine which class they belong to, either phenolic acids (non-flavonoids), flavonoids, stilbenes, or lignans [41].

The functions of polyphenols in biological reactions and physiological processes are also wide-ranging. They have been well-investigated to confer numerous health benefits, especially in curbing the global health burden of non-communicable diseases (NCDs) by primarily acting as antioxidants. Antioxidants have pharmacological properties such as anti-inflammatory, anti-ageing, and anticancer, thus playing crucial roles as health-protecting compounds. They work by breaking harmful chain reactions and excessive cell proliferation occurring in our body through the actions of unstable molecules called free radicals and pro-oxidants. Antioxidant molecules donate some of their electrons to neutralize and inactivate the free radicals from damaging other body cells, leading to the pathogenesis of many chronic diseases [42]. Table 1 presents the major classes of polyphenols, selected polyphenolic compounds, the health benefits they render, and sources from underutilized seeds.

The agricultural and food-processing industries produce a huge number of unused by-products that can be spared and transformed into high-quality food products for the benefit of both animals and humans. In recent years, the global attempts to valorise agricultural wastes have been soaring as there has been a growing body of research that shows that polyphenols are also concentrated in agricultural residues such as fruit peels and seed meals. When consumed correctly and not reaching toxic levels, these bioactive compounds have essential roles in disease prevention, maintenance, and treatment [59]. Thus, they are relevant to our daily diet for optimal well-being. Despite that, studies on the bioavailability of polyphenols in whole plant-based foods are still scarce, and the metabolic fate of these antioxidants in the body is not fully known. Polyphenols are isolated and purified from plants (fruits, vegetables, and agricultural by-products) and transformed into supplements [39]. Hence, acquiring optimum health benefits from polyphenols in the food matrix would require us to eat a balanced, moderate, and varied diet [60,61].

Plant seeds are one of the richest sources of polyphenols, either concentrated in the seed coat or the cotyledon or both. Interestingly, several seeds have been labelled as “specialty seeds” or “super seeds” due to their remarkable biological activity, attributed to high levels of bioactivity and bioavailability. These tiny seeds are high in polyphenols and rich in monounsaturated fats, protein, vitamins, minerals, and fibre. Black cumin, chia, hemp, flax, perilla, pumpkin, quinoa, and sesame seeds are the specialty seeds that have been reported to be the most typically utilized in human diets for generations [62]. Furthermore, some of these seeds are included in the dietary guidelines of nations such as the United States, Australia, and Qatar as part of the healthy dietary selection [63,64,65]. Seeds are versatile because they may be consumed in several ways, including appetizers, cuisines (e.g., incorporated into cereals, salads, and various main meals), and food products such as bread and spreads. Other plant seeds and polyphenols detected in plants are summarized in Table 1.

## 4. Seeds from Fibre Crops That Are Potential Sources of Polyphenolic Compounds

Okra, cotton, hemp, and kenaf plants are some examples of promising sources for natural polyphenols. They have been described as equally crucial as sustainable sources of natural fibre with many potential uses [66]. However, the industrial fibre processing from these plants generates a large number of waste materials, including seeds that can alternatively be recovered and utilized for the addition of values (nutritional qualities, oxidative stability, and sensory properties), instead of being discarded [2,13,30]. Today, many studies have demonstrated the widespread food applications of these seeds and their derivatives, attributable to the significant dietary constituents (protein, healthy fats, dietary fibre, vitamins, and minerals) essential for many bodily processes, as well as numerous dietary polyphenolic compounds that confer physiological benefits beyond essential nutrition [13].

### 4.1. Okra (Hibiscus esculentus *L*.)

#### 4.1.1. Origin, Cultivation, and Uses of Okra Plant

Okra (*Abelmoschus esculentus* or *Hibiscus esculentus* L.), also recognized as ‘lady’s finger’, is a commonly consumed vegetable worldwide. Exploring the history of okra cultivation, it began in Ethiopia (East Africa) and Egypt many centuries ago. Currently, okra planting has spread to other regions where the temperature is also warm, particularly Africa, Asia, Southern Europe, and America [67].

Almost every component of the okra plant is favourable for the benefit of humankind. Traditionally, okra leaves and flowers can be made or added into soup and stews because of their slimy property that contributes as a thickening agent. On the other hand, okra fruit as a whole can be eaten raw or steamed and added with some seasonings to mask the bland taste and bring some extra flavours [67]. Industrially, bast fibre from its stem is of great value for producing eco-friendly biocomposites [68]. Okra seeds can be found within the chamber-like pentagonal pods of the green-coloured fruit [69]. The seeds are white and often eaten together with the fruit. It has been investigated that the seeds of okra, which constitute about 17% of the whole vegetable, contain the highest nutritional content [70].

Okra has exceptional nutritional properties, yet it has a poor economic value in some parts of South Africa. The crop is exclusively grown by small local farmers who usually lack understanding about the influence of its nutrients when used in locally produced food products. Furthermore, local farmers lack enough expertise on the best methods for producing varieties and maximizing output for specific technological uses [71,72]. Okra pods are often exploited for their pectin content for food applications (as thickeners, emulsifiers, and stabilizers), leaving the seeds as waste [73]. The leftover seeds may be processed further and incorporated into various well-known foods containing okra seed, helping drive food innovation initiatives and food loss management [72].

#### 4.1.2. Utilization of Okra Seeds as Food Ingredients

Usage of okra seeds as a part of the human daily diet is wide-ranging. The seeds can be roasted and ground to become a caffeine-free coffee-like powder, popular among Turkish people [74]. As described in the literature, okra seed coffee has an indistinguishable taste from regular coffee, benefiting people intolerant of caffeine’s adverse effects [75].

Edible flour made from okra seeds could be a great substitute or replacement to wheat because it can offer sufficient protein, as wheat flour does, and it contains high dietary fibre [76]. Ofori et al. [76] developed seed flours from two variants of okra seeds, which retain high protein (16.8–17.4%) and fat (47.8–48.0%) contents. Furthermore, blending okra seed flour with wheat flour can also contribute to the higher fibre content in the baking ingredient, as demonstrated in the study by Rindiani and Kumalasari [77], where they produced steamed cake from the okra-wheat flour mixtures with enhanced nutritional properties. The steamed cake’s highest fibre content (5.34%) was formulated with 50% okra seed flour [77]. Interestingly, okra seed flour produced via steam explosion by Hu et al. [78] demonstrated its promise as a gluten-free food ingredient, suitable for consumption by people who cannot tolerate gluten (i.e., patients with gluten intolerance and celiac disease).

In another study, Omoniyi et al. [79] prepared carbohydrate-based soups fortified with okra seed flour acceptable to the sensory panellists within the fortification range of 4–12% (*w*/*v*). The improved recipe benefits from energy supply and mineral replenishment, and especially iron, as the primary mineral in okra seed flour (0.032 g/100 g).

Okra seed powders that have been defatted, such as okra seed meal, okra seed protein concentrate, okra seed protein isolate, and okra seed protein hydrolysate, are protein-rich with excellent functional properties, making them suitable as a functional food to be applied in beverage and bakery products [80]. Noteworthy findings revealed from the study included more excellent water absorption at increased temperature, improved foaming ability and solubility at higher pH by all samples, and great in vitro digestibility of protein and antioxidant capacities by protein hydrolysate.

#### 4.1.3. Nutritional Properties and Polyphenolic Contents of Okra Seeds against Diseases

Seeds of okra are made up of 20–35% protein content with reasonable amounts of amino acids comparable to those of soybeans. The Protein Efficiency Ratio (PER) of okra seeds is remarkably better than soybeans [80,81,82]. The oil content in okra seeds is almost similar to other oilseeds, which is in the range of 20–40%, and the oil abundantly consists of PUFA such as linoleic acid. The seeds are also an essential source of phenolic constituents such as oligomeric catechins (2.5 mg/g of seeds) and flavonol derivatives (3.4 mg/g of seeds) [70,83].

Okra seeds have been claimed to deliver beneficial health effects in many studies, as they have been found to have potential antidiabetic, antifatigue, antistress, and anticancer activities due to their generous amount of numerous phytochemicals [83,84,85,86]. Those health-beneficial phytochemicals include isoquercitrin and quercetin-3-O-gentiobiose [85]. The potential of okra seed extract as an antidepressant was scientifically proven in Xia et al. [87] investigating male ICR mice. In the experiment, mice treated with aqueous okra seed extract up to 600 mg/kg body weight underwent a series of behavioural tests. They showed positive results in suppressing depression signs, namely hypothalamic–pituitary–adrenal (HPA) axis hyperactivity, oxidative stress, and imbalance of neurotransmitter levels in the hippocampus and frontal cortex of the brain. In support of these outcomes, applying UPLC-DAD/Q-TOF MS technology identified various phenolic compounds in okra seed extract and quantified them, constituting almost 30% of the extract. The main compounds were identified as catechin and quercetin derivatives. Therefore, it can be concluded that the antidepressant activity of okra seed extract might be due to the antioxidant activities of these flavonoids, which assist in reducing the effects of oxidative damage in the brain [87].

**Figure 2:**
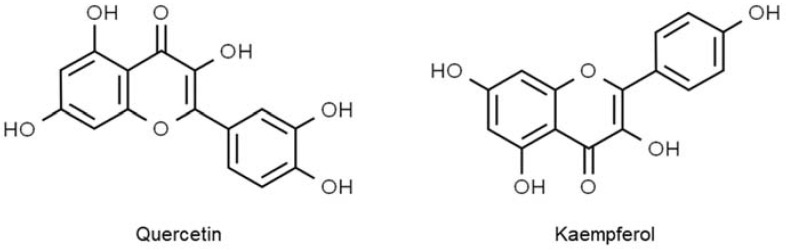
Polyphenolic compounds in okra seed.

Evidence strongly supports the notion that dietary polyphenols are beneficial in treating diabetes mellitus. Due to the excellent composition of polyphenolic compounds in okra seed extract, its antidiabetic property has also been investigated and confirmed in many studies. By employing in vitro α-glucosidase enzyme inhibition assay, okra seed extract was observed to confer an equivalent antidiabetic effect with acarbose, an antidiabetic medication [88]. The efficacy of okra seed extract as an inhibitor to α-glucosidase activity could dampen carbohydrate digestion into glucose, thus lowering postprandial glucose and insulin levels in diabetic patients [89]. Among other polyphenols that were detected in okra seed extract, derivatives of quercetin (quercetin 3-O-(malonyl)-glucose and quercetin-3-o-glucose-xylose) were the ones with the highest concentrations, indicating that the antidiabetic effect in the study was explicitly contributed by these compounds (Figure 2) [89].

In the study by Ong et al. [88], polyphenol-rich extract from okra seed also has been demonstrated to be potentially vasoprotective. The promising vasoprotective effect of okra seed extract was achieved via several mechanisms. Quercetin in okra seed extract exhibited strong antioxidative and cytoprotective effects towards endothelial cells (HMEC-1) from the reactivity of hydrogen peroxide radicals, thereby improving impaired endothelial function. In addition to that is relief from vascular inflammation induced by an inflammatory cytokine, tumour necrosis factor-α (TNF-α). Inhibition of TNF-α efficiently reduced the expression of genes of two cell adhesion molecules, vascular cell adhesion molecule and E-selectin, which usually increased in untreated inflammation. As previously reported, quercetin also impeded the expression of other inflammatory factors, which are interleukin-1β (IL-1β) and interleukin-6 (IL-6) in vitro [90]. Prevention of vascular oxidative stress can decrease the likelihood of contracting the risk factors of cardiovascular diseases such as hypertension, hyperglycaemia, and hypercholesterolemia, to name a few. As okra seed extract is antioxidative, vasoprotective, and possibly cardioprotective, it may be helpful as a potential complementary and alternative treatment in managing cardiovascular diseases. Many traditional societies and cultures have utilized okra and its parts for health maintenance and disease treatments [91].

Uniquely, the intake of okra seed extract containing high levels of polyphenols can also produce an antifatigue effect in addition to its known capability as an antioxidant source. In one study, it was reported that okra seed extract showed highly remarkable in vitro antioxidant activities as measured through DPPH, FRAP, and reducing power assays as compared to extracts from okra pods and skins [85]. Further, it was elucidated that the antifatigue property of okra was also contributed by its seeds, where polyphenols, namely isoquercitrin and quercetin-3-O-gentiobiose are found ubiquitously, in comparison to other okra constituents. The parameters associated with oxidative stress-related fatigue, such as levels of blood lactic acid (BLA) and blood urea nitrogen (BUN) and liver levels of malondialdehyde (MDA), are reduced considerably following okra seed extract treatment up to 0.6 g/kg body weight for 21 consecutive days in male ICR mice. On the other hand, hepatic glycogen (HG), total superoxide dismutase (SOD), and glutathione peroxidase (GSH) levels positively increased. Hence, these are the possible mechanisms on how quercetin and its derivatives in okra seed extract can mitigate fatigue, the results of which are similar to those found in a previous study [92]. Moreover, recent literature also demonstrated that quercetin exhibits good antifatigue capacity through the enhancement of antioxidant activities, glycogen storage, and muscle function of male BALB/c mice after 6 weeks of 0.005% quercetin supplementation [93].

### 4.2. Cotton (Gossypium *spp*.)

#### 4.2.1. Origin, Cultivation, and Uses of Cotton Plant

The cotton plant or *Gossypium* spp. is a flowering plant from the Malvaceae family, said to have originated from tropical and subtropical regions of Africa, South America, and Asia in the past 70 centuries. Primarily, cotton plants of *G. hirsutum*, *G. barbadense*, *G. arboreum*, and *G. herbaceum* species are cultivated mainly for their natural fibre, which is helpful in the textile manufacturing and apparel industry. According to Rathore et al. [94], the major countries that produce cotton plants are India, Pakistan, and China, which suffer significantly from malnutrition issues in their population. Cotton is undoubtedly one of the most profitable cash crops that could boost income for millions of lives, notably in low-income nations, by providing opportunities for employment and sales [95,96].

The cotton plant is helpful for many daily life purposes. Cotton buds are the most often used component of the plant. A variety of goods, including but not limited to textiles, edible oil, and paper, can be produced from cotton buds as a raw material [97]. Cotton fibre is among the most significant natural textile fibre in the world. It has soothing and cooling properties, providing comfort and absorbency. Not only that, but cotton fibre is currently also, among other bast fibres, favoured for automotive uses because of its lightweight, renewable, and biodegradable attributes. Natural fibre composites are relatively new in the electrical, electronics, and sports industries, but they could acquire a significant market share [66].

Massive amounts of agricultural waste are yielded from the cultivation of cotton. They are helpful in developed nations but tend to be overlooked in developing countries due to a lack of waste valorisation expertise and technology. There are two categories of cotton production waste which have been established, depending on how they are reused after being discarded: on-farm (for agricultural uses) and off-farm (for industrial uses). Cotton seeds belong to the off-farm waste category, which is the by-product of cotton after ginning process, and account for merely 20% of the crop value [98]. Whole cottonseed and cottonseed meal are among the common feed for livestock because of their affordability, being easily obtainable, and having high-quality protein and fat contents, which are required for good animal performance [99]. Cottonseed meal is often utilized in cattle feed; however, its usage in human nutrition is limited because of the presence of gossypol as a limiting factor, understood to be toxic to monogastric animals. Despite this, numerous solutions have been explored to detoxify cottonseed so that other essential nutrients from cottonseed such as protein, essential fatty acids, and antioxidants, among others, can be utilized to their full potential [100,101]. According to the United Nations Food and Agriculture Organization and the World Health Organization, the acceptable levels of gossypol in cottonseed products are 450–600 ppm (free gossypol) and 12,000 ppm (total gossypol) [102]. Many well-researched strategies, including genetic engineering [103], enzyme detoxification [104,105], fermentation, and solvent extractions have been applied to eradicate the gossypol-containing glands from cottonseed.

#### 4.2.2. Utilization of Cotton Seeds as Food Ingredients

Apart from polyphenolic compounds, other bioactive compounds, such as β-sitosterol and tocopherols, are also abundant in cottonseed, particularly in their oil content [106,107]. Because it is high in saturated fats, cottonseed oil can be an excellent choice for deep frying purposes, which gives the fried foods a nutty or buttery aromas. Lack of linolenic acid and high levels of potent antioxidants (i.e., tocopherols) also contribute to the stability and suitability of cottonseed oil as a frying oil [108].

Before the acceptable limits for gossypol content were defined, cottonseed meal or flour was rarely used for human consumption despite its high protein content (30–50%) [109,110]. Instead, it is more often utilized for livestock, specifically for adult ruminants, because of their digestive systems’ ability to detoxify the polyphenolic compound [111]. Cottonseed flour can be detoxified from free gossypol and total gossypol up to 99.3% and 89.2%, respectively. As a result, cottonseed flour now can be widely utilized as a protein-rich food ingredient (e.g., incorporated in baked goods, pasta, and protein bars) for human consumption especially in nations where the severe protein energy malnutrition (PEM) problem needs to be tackled [100].

Kumar et al. [112] found that cottonseed protein isolate (CSPI) made from defatted cottonseed meal, meeting the essential requirements following Food Safety and Standards Regulations (FSSR), 2011, can be regarded as a functional food. CSPI has a crude protein of 93.1%, comparable to that of soybean protein isolate (SPI) (95.4%), and only trace amounts of total gossypol and free gossypol. On top of that, CSPI is also better than SPI concerning oil holding and foaming capacities. These remarkable nutritional and functional properties resulted from extracting under an alkaline condition [112].

In Southern India, a nourishing drink made from cottonseed called ‘Paruthi Paal’ has been traditionally consumed for various therapeutic purposes, predominantly in treating fever symptoms [107]. Paruthi Paal or cottonseed milk resembles cow’s milk and is rich in protein. Using glandless cottonseeds is recommended in order to optimize protein utilization. Reduction of gossypol content can also be achieved during the milk-like extraction process. Under optimized conditions, Subramani et al. [113] obtained safe and nutritious cottonseed milk with gossypol content diminished by 37.14% through the conventional extraction method, whereas protein content reached 21.35% through enzyme-assisted aqueous extraction. Other innovative products made from cottonseed milk could also be developed, such as tofu, yoghurt, and cheese, to provide more attractive healthy food options. Nevertheless, other health benefits such as antihypercholesterolemic, anticancer, and antihypertensive claimed to be associated with cottonseed milk call for more scientific investigations [107].

#### 4.2.3. Nutritional Properties and Polyphenolic Contents of Cotton Seeds against Diseases

There are two types of fibre which wrap the seeds of the cotton plant in a capsule called a ‘boll’, and a ‘ginning’ procedure can separate them. Cotton seeds have various uses as food due to their remarkable physicochemical and nutritional properties. Cottonseed protein is considerably high (20–25%). Its quality is outstanding, as measured by the amino acid composition and numerous functional properties. It was indicated that glandless cottonseed protein is nearly comparable or even better than that of soybean protein [101,114]. Besides, the oil of the seeds (17–22%) is composed of fatty acids, namely palmitic acid (22%), oleic acid (20%), and linoleic acid (54%) [106].

Gossypol is known to be toxic to the liver and reproductive organs [115]. Nevertheless, many studies have also reported its desirable bioactivities towards health as an anti-inflammatory, anti-obesity, and antifungal agents, and as an anticancer drug for several cancer types [116,117,118,119,120,121]. Promising anti-obesity activity of gossypol was exhibited in female laboratory rats, in which their body weight gain and appetite were curbed after 5 and 10 mg/kg body weight/day of gossypol treatment for 15 days [122]. Further, in an in vitro assessment, cottonseed oil enriched with gossypol was also proven to control proliferation and adipogenesis of human pre-adipocytes. These positive health outcomes were attained through reductions in a few gene expressions, intracellular triglyceride level, and glycerol 3-phosphate dehydrogenase (GPDH) activity [117]. Additionally, gossypol portrayed its potential in eliminating fungal pathogens through some possible modes of action. Two of these actions work by preventing fungal access to carbon and nitrogen, consequently inhibiting their cell wall formation, and obstructing the development of aflatoxisomes [118]. On the other hand, gossypol performs potential cancer-fighting benefits by several vital processes such as altering a specific gene expression and inhibition of DNA synthesis so that cancer cells cannot proliferate [123,124].

**Figure 3:**
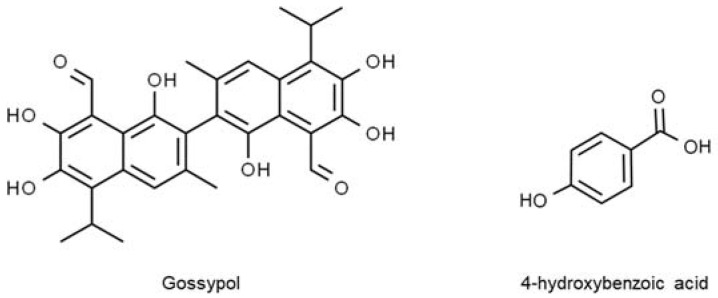
Polyphenolic compounds in cotton seed.

Ideally, glandless cottonseed devoid of dark spots (pigment glands) and the presence of gossypol exhibit safer and better health effects than glanded cottonseed. Moreover, other polyphenolic compounds with scientifically proven health benefits such as gallic acid, quercetin, flavonol glycosides, and 3,4-dihydroxybenzoic acid co-exist in cottonseed (Figure 3). Due to that, gossypol detoxification is essential so that the role of these polyphenols as free radical scavengers in the prevention of many chronic illnesses can be maximized [98]. In one study involving in vitro assays, ethanolic extracts of cottonseed either with or without glands were non-deleterious towards mouse macrophages and adipocytes. In addition, glandless cottonseed extract also showed great potential as an anti-inflammatory agent by stimulating an RNA-binding protein, tristetraprolin (TTP), to inhibit inflammation in the cells [116]. Therefore, glandless cottonseed, which is as vital as other oilseeds such as soybeans and sesame seeds, can be fully utilized and developed into various food products and would consequently help stimulate economic growth in cotton-producing countries [94,95,96].

### 4.3. Hemp (Cannabis sativa *L*.)

#### 4.3.1. Origin, Cultivation, and Uses of Hemp Plant

*Cannabis sativa* L. is a flowering plant from the Cannabinacae family, originating from Central Asia. Before the legalization of its cultivation, hemp was replaced by cotton and flax as the primary oilseed commodities in many countries [125]. At present, it has been widely cultivated in France, Russia, China, Chile, Canada, and the United States [126,127]. This is due to its safety and high-quality properties to be a source of natural fibre (from its long stems), oil (extracted from the seeds), foods, as well as medicine [128]. The history of usage of hemp seeds as food in China, Australia, and Canada have dated back to around 20 years ago, and recently in the United States and India after the THC level in hemp has been modified to less than 0.3% [128,129,130]. Since then, the development of food products from hemp has been made possible [128].

More than 40 hemp cultivars have been documented, with Finola being the most often grown for commercial applications [131]. In addition, *C. sativa* may also be divided into two types: drug (marijuana) and non-drug (hemp). The drug variety is often used for therapeutic and recreational uses. Conversely, the non-drug variety is intended for food and fibre uses [125]. Fibre from hemp stems is used in the textile, construction, and automotive sectors, and as a bedding material for livestock owing to its exceptional qualities (i.e., light, robust, high absorption capacity, and good antibacterial activity) [132]. Hemp leaves and flowers contain bioactive chemicals integrated with medications and human diets and drinks [133]. Hemp leaves have also been fed to livestock, particularly ruminants, as an appetite stimulant to improve food intake, growth, and reproduction [134].

Hemp seeds are contained within a hard shell for protection and coated with two-layered pericarps. They are characterized by dark red to brown colour, darker-shaded stripes, and approximately 2.5 to 5.0 mm lengths. Hemp seeds are also regarded as the fruit of the *C. sativa* plant, constituted by an endosperm and two cotyledons on the inside that comprise the embryo [135,136]. In comparison to the stem and the leaves, hemp fruits or seeds are often underappreciated, although they are the main edible parts and a powerhouse of nutrients and phytochemicals with disease-fighting capabilities [128].

#### 4.3.2. Utilization of Hemp Seeds as Food Ingredients

Hemp seeds can be consumed in various forms; raw, roasted, or in the forms of oil and milk extracted from them [137]. Roasted and ground hemp seeds are sold as a snack by street vendors in China [125]. By tradition, crushed hemp seeds are mixed with ingredients such as herbs and spices to become a smooth paste which Nepali and Indian people would eat together with rice [136].

There are also hemp seed-derived food products such as protein powder, flour, and butter, which have been derived due to their high protein content of approximately 25% [125,138]. Improved nutritional values and sensory acceptability have been demonstrated in the development of food products such as ready-to-eat snack, energy bar, and gluten-free bread supplemented with defatted hemp cake, hemp flour, and hemp protein concentrate [139,140,141].

Hemp seed oil is also in high demand because of its high quantity of unsaturated fatty acids (over 90%) and its optimal ratio of ω-3 to ω-6 fatty acids that have beneficial effects on heart health [142]. Cold-pressed hemp seed oil has a distinctive flavour that is reminiscent of walnuts and sunflower seeds. It has the potential to be a dietary supplement for the treatment of atopic dermatitis if consumed regularly [143]. In some parts of the world (e.g., Russia) where animal-derived butter and margarine are poorly available and affordable, hemp seed oil has become a replacement ingredient to produce this semi-solid food product [125].

In Canada and some European countries, commercial hemp milk beverages are readily accessible. Even after numerous steps in the process of extracting the milk-like solution from hemp seeds, all nutrients in the liquid beverage form are retained. According to reports, hemp-based beverages contain 4% and 5% protein and fat, respectively [144]. Chichłowska et al. [145] studied the biological effects of hemp milk. After 21 days of consumption, hemp milk revealed notable hypocholesterolemic benefits (reduced serum total cholesterol and triglycerides) and influence on thyroid hormones in Wistar rats.

#### 4.3.3. Nutritional Properties and Polyphenolic Contents of Hemp Seeds against Diseases

Hemp seeds have high nutritional and commercial values due to their protein, fatty acid, carbohydrate, fibre content, and therapeutic compounds. Hemp seed contains a good amount of protein, between 25% to 30%, with an amino acid profile close to soybean and egg white proteins. Despite its protein quality, much attention is given to hemp seed oil [135]. Similarly, oil content in hemp seeds is about 25–30%, and it is rich in unsaturated fatty acids such as oleic acid, linoleic acid, α-linolenic acid, γ-linolenic acid, and stearidonic acid, which is reported to be more than 90%. Moreover, hemp seed has the ideal ratio of ω-6 to ω-3 fatty acids (between 2:1 and 3:1) to prevent many chronic ailments such as heart disease, cancer, and rheumatoid arthritis [142]. On the other hand, saturated fatty acids, namely palmitic acid and stearic acid, are present only in meagre amounts compared with other oilseeds [125].

**Figure 4:**
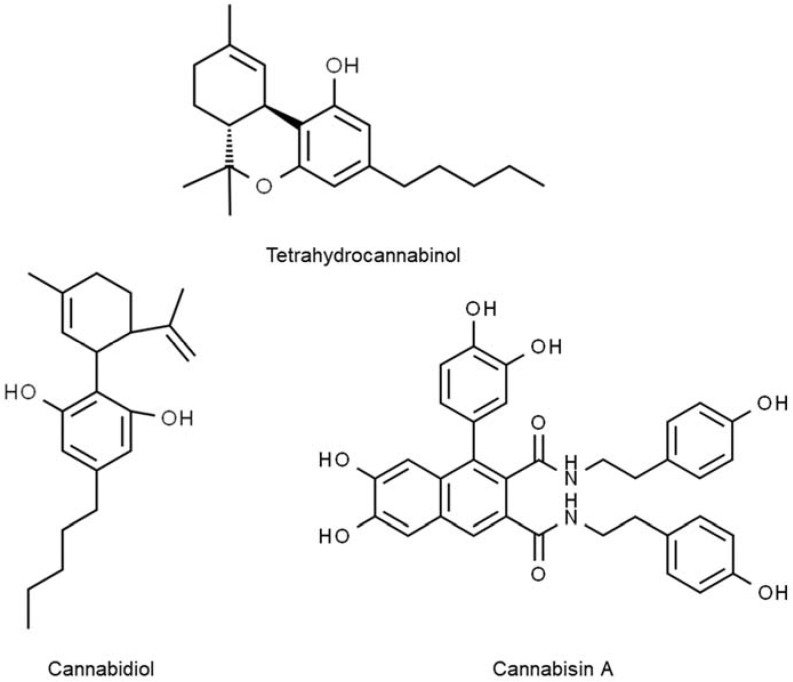
Polyphenolic compounds in hemp seed.

A group of chemical compounds that could give rise to psychoactive or hallucinogenic effects to people consuming it, identified as cannabinoids, are found in the flowers and fruits of hemp. Cannabinoids are terpene phenolics, a combination of terpenes and phenolic compounds exclusively synthesized in *C. sativa* [135]. These compounds are also present in hemp seeds but in relatively smaller quantities than in the flowers and fruits. The first primary prevalent type of cannabinoids in hemp seeds is ∆-9-tetrahydrocannabinol (Δ-9-THC), as shown in Figure 4. It is one of the commonly used drugs worldwide for medicinal purposes of alleviating stress and depression. However, Δ-9-THC can be intoxicating with excessive and long-term use. An enormous controversy centres around the human consumption of hemp seed and its derived food products. As a result of advances in food technology, hemp seed cultivation and consumption have become legal in countries such as Australia, Canada, and the United States, provided that the THC level is below 0.3% *w*/*w* [128]. The other prominent cannabinoid, cannabidiol (CBD), is non-psychoactive. Due to its polyphenolic nature and potent antioxidant activities, CBD has been declared safe and used to treat brain disorders such as epilepsy. Epilepsy is characterized by unusual brain activity, repeated seizures or erratic behaviour episodes, and reduced awareness. The roles of CBD as an antiepileptic agent involve controlling inflammation reaction, preventing nerve damage, and regulating neurogenesis in the brain [146]. Furthermore, Gray and Whalley [147] suggested that CBD can curb epileptic symptoms by antagonizing the G protein-coupled receptor-55 (GPR55) receptors, suppressing Transient receptor potential vanilloid-1 (TRPV1) receptors, and blocking adenosine transport into cells.

Other than flavonoids and phenolic acids, the most abundant polyphenols, lignanamides, are also found in hemp seeds. Lignanamides are a subclass of lignan and are a highly potent antioxidant. In earlier scientific reports, the total lignanamides represented by hemp seed is 77 mg/100 g dry weight and they are mostly composed of cannabisin A, cannabisin B, and cannabisin M [148,149]. A laboratory study reported that lignanamides isolated from hemp seed possessed the ability to block the action of acetylcholinesterase [150]. The role of this enzyme is to break down acetylcholine that functions as a critical neurotransmitter in both central and peripheral nervous systems. Therefore, to treat neurodegenerative disorders such as Alzheimer’s and Parkinson’s diseases, balanced levels of acetylcholine can be maintained through acetylcholinesterase inhibitors. Lignanamides are various pharmacologically active compounds extracted from natural sources tested against acetylcholinesterase activity [151]. Moreover, Irakli et al. [152] pointed out that the antioxidant capacities of hemp seed extract are mainly contributed by polyphenolic compounds instead of other antioxidative compounds such as tocopherols and carotenoids.

### 4.4. Kenaf (Hibiscus cannabinus *L*.)

#### 4.4.1. Origin, Cultivation, and Uses of Kenaf Plant

Kenaf (*Hibiscus cannabinus* L.) plant is a common wild plant native to subtropical and tropical regions of Africa and Asia. It has other names in other places, such as ‘ambari’ in Taiwan and ‘hong ma’ in China [153]. The first cultivation of the kenaf plant was in Egypt and Africa, dating as far back as 3000–4000 years ago. Until today, kenaf has been widely cultivated in India, China, Bangladesh, the United States of America, Indonesia, Malaysia, South Africa, Vietnam, and Thailand [154,155].

**Figure 5:**
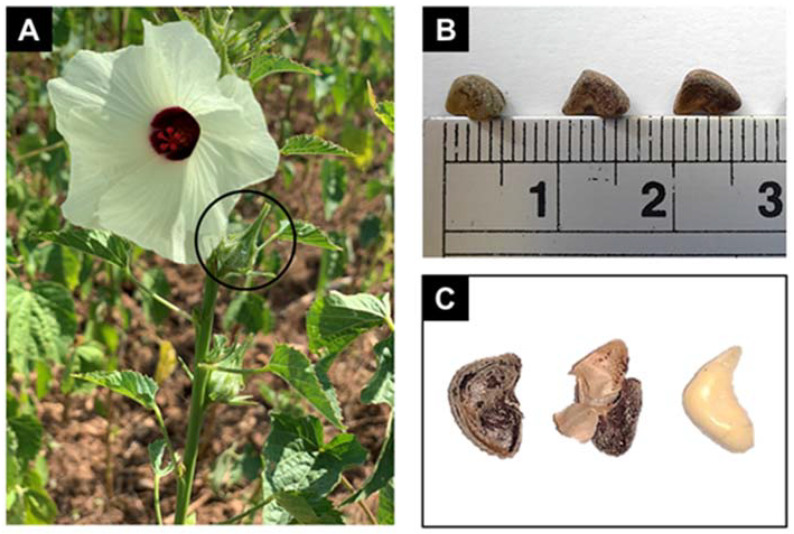
Kenaf plant, kenaf seed size measurement, and kenaf seed components. (**A**) A young kenaf plant with a flower, stem, seed pod, and shoots. (**B**) Measurement of kenaf seed length using a standard-sized ruler. (**C**) From the left are whole kenaf seed, seed hull, and dehulled seed.

Kenaf plants can grow up to 3.5 m tall. The diameter of its stem is between 1.0 to 2.0 cm, its leaves come in various shapes and are about 10 to 15 cm long, and the bell-shaped flowers can be a white, yellow, or purple in colour. Its leaves are widely consumed as a vegetable, made into beverages, produced into flour for baking, and transformed into traditional herbal medicine by locals in India and Africa [156,157,158,159]. Birhanie et al. [160] also revealed that kenaf leaf extracts could be a source of natural antioxidants and are particularly useful as natural microbial agents or bio preservatives, and are effective in inhibiting food spoilage and harmful microbes. On the other hand, kenaf seeds are formed in seed pods (about 1.9–2.5 cm long and 1.3–1.9 cm diameter) after pollination. The seed pods are pointed, oval, and covered with fine hairs. About 20 to 26 black, wedge-shaped kenaf seeds can be obtained (Figure 5). The size of the seeds is approximately 0.5 to 0.6 cm long [161].

Kenaf seeds are the principal by-product of the kenaf plant, but they are considered low in economic value [162]. As reported previously, about 98% of harvested seeds from kenaf planted are discarded. It only requires 2% of the total 1000 kg/hectare seed output needed to plant a hectare of a new generation of kenaf plants [163]. Disposing of wastes can be damaging to the environment. In response to the problem, wasted or abandoned kenaf seeds can be reused by being transformed into value-added goods with high-value benefits, including food products. The seeds are edible and can be made more palatable by imparting food additives and seasonings (e.g., sugar, salt, acids, herbs, and spices) to enhance their earthy flavour [164]. Kenaf seeds have been studied in several animal experimentations and have been found to be non-toxic and safe for consumption [165,166]. Therefore, they could provide multipurpose uses such as food fortification, dietary diversification, and medicinal purposes [167].

#### 4.4.2. Utilization of Kenaf Seeds as Food Ingredients

In the same manner as soybeans, many food products, such as edible oil, tofu (obtained from seed milk), tempeh (derived from fermented seeds), flour, and protein powder (procured from defatted seed meal), can be attained from kenaf seeds. There has been a lot of study and development of food products developed from kenaf seeds in recent years. Because kenaf seeds have a high-fat content, vegetable oil can be extracted via solvent extraction or supercritical fluid extraction. According to a study, the composition of kenaf seed oil (KSO) is nearly identical to that of cottonseed oil [168]. As a result, it has much potential as a food oil, such as cooking oil and margarine [169]. Vitamin E, β-sitosterol, α-linolenic acid, and ω-3 fatty acids are among the bioactive substances found in KSO, which have a variety of health benefits [170]. Natural antioxidants can also assist in halting oxidation and extending storage stability, and therefore preserving the quality of fats, oils, and lipid-containing foods [23].

Defatted kenaf seed meal (DKSM) can be obtained from the processing of KSO. Protein (26.19/100 g DKSM), carbohydrate (57.09/100 g DKSM), total phenolic (3399.37 µg GAE/g defatted material), and flavonoid content (251.00 mg RE/g defatted material) are indeed high in DKSM [136]. Owing to its high protective effect against oxidative stress and inflammation in rats with hypercholesterolemia, DKSM has been proven to have good potential as a functional ingredient in foods [171]. The above-mentioned positive health effects are attributed to two bioactive components found in DKSM: phenolics and saponins [171].

Moreover, kenaf seed protein concentrate (KSPC) and kenaf seed protein isolate (KSPI) can be produced from DKSM because of their valuable high protein content and rich essential amino acids [172,173]. Protein concentrates and isolates from kenaf seeds are both high-quality components that can be included in various food products (e.g., condiments, bakery, and confectionery products) and supplement formulations (e.g., protein shakes), particularly because of their high-protein content, excellent oil and water absorption capacities, and exceptional foaming capacity [172,173]. Additionally, KSPC and KSPI have the potential as the main ingredient to produce meat analogues such as tofu, tempeh, and textured vegetable protein [173].

Kenaf seeds can also be used to make a milk-like solution, also known as ‘kenaf seed milky extract’ or ‘plant-based kenaf seed milk’ that can serve as a nutritional beverage. A slurry is formed when soaked seeds are crushed with excess water quickly. The use of kenaf seed milk extract in food products is receiving considerable attention. Several experiments have been undertaken to generate novel food products from kenaf seed milk extracts, such as tofu made from curdling and kenaf milk beverages with appealing flavours [164,174].

#### 4.4.3. Nutritional Properties and Polyphenolic Contents of Kenaf Seeds against Diseases

Kenaf seeds have a tremendous nutritional composition, bearing reasonable amounts of macronutrients, micronutrients, and bioactive constituents. It is high in carbohydrate (23.2–33.10%), crude protein (21.4–30.5%), and fat (18.9–24.8%) contents [169,172,175]. Among major minerals in kenaf seeds are calcium, magnesium, potassium, and phosphorus, essential for many biological and cellular functions [166,169]. Additionally, it is profound that kenaf seed oil has a comparable fatty acid composition to cottonseed oil, with primary fatty acids being palmitic, oleic, and linoleic acids [168,176]. Kenaf seed has also been described as a potential source of dietary fibre that can offer different applications in the food system [177].

**Figure 6:**
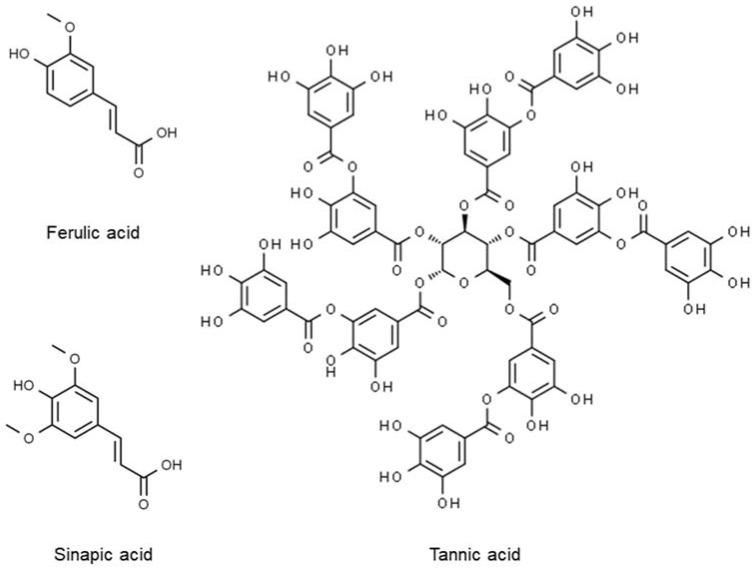
Polyphenolic compounds in kenaf seed.

The presence of different phytochemicals, including polyphenols, plant sterols, essential oil, and essential fatty acids, allows it to be a food with functional capabilities to improve and maintain health and treat diseases [176,178,179]. Polyphenols in kenaf seeds consist of a diverse range of compounds. Many studies demonstrated that aqueous or water extracts of kenaf seed are very rich in phenolic antioxidant compounds, suggesting that most phenolic compounds in kenaf have highly polar chemical structures that include one or more hydrophilic hydroxyl groups [179,180,181]. Kai et al. [182], in their study, reported the presence and quantified levels of tannic acid (2302.20 mg/100 g), sinapic acid (1198.22 mg/100 g), catechin hydrate (502.73 mg/100 g), and 4-hydroxybenzoic acid (255.84 mg/100 g) in kenaf seed extract (KSE) as the significant phenolics, among others (Figure 6). Tannic acid has been demonstrated to have antioxidant, antidiabetic, anticancer, antibacterial, and anti-inflammatory activities and, thus, possess a spectrum of uses [183,184,185]. Sinapic acid, too, has already been evaluated pharmaceutically for its powerful antioxidant, anti-anxiety, antilipidemic, and neuroprotective qualities [186]. The 4-hydroxybenzoic acid is a phenolic derivative from benzoic acid that possesses lipid-lowering (by inhibiting hydroxyl-3-methylglutaryl-coenzyme A reductase (HMGR) activity and oxidative stress and increasing faecal acidic sterol excretion) and hypoglycaemic (by stimulating peripheral glucose utilization, a similar property to insulin) efficiencies [171,187,188]. Besides these predominant components, kenaf seed also contains gallic acid, syringic acid, vanillic acid, p-coumaric acid, and protocatechuic acid [179,182,189].

Antioxidative polyphenolic compounds are known to exert health benefits by alleviating oxidative stress, a condition where many free radicals damage normal tissues. There are many research articles in the literature pertaining to the antioxidant properties of kenaf seed through many antioxidant assays. The kenaf seed extract (KSE) obtained from ultrasonication in the study by Nyam et al. [190] contains 170.72 mg GAE phenolics per 100 g of sample using a spectrophotometric method. A prior study reported a higher total phenolic content in KSE with a value of 5880.56 mg/100 g, using high-performance liquid chromatography (HPLC) [182]. The total phenolic content in kenaf seed aqueous extract was 162.7 mg TAE/100 g and 750 mg GAE/100 g in more recent studies by Ryu et al. [179] and Adnan et al. [181], respectively.

In the past several years, much research using tissue cultures and organisms has documented various health effects that humans can potentially benefit from consuming kenaf and kenaf seed-based food products. KSO was shown to exhibit cytotoxic effects against human breast and colon cancer cell lines due to its phenolic contents [141,161]. In the study by Foo et al. [165], cytotoxicity of KSO against leukaemia cells was also evident in both in vitro and in vivo methods. Hence, kenaf seed could be a potential alternative medicine in treating many cancers known to be life-threatening [191].

Additionally, based on an in vivo study using a hyperlipidemic rat model, kenaf seed extract (KSE) significantly reduced total serum cholesterol, atherogenic index (AI), and coronary risk index (CRI) by supplementing KSE in the animal diet at dose 400 mg/kg body weight for 32 days [182]. The specific mechanism postulated to clarify the heart-protective effect of antioxidant-rich KSE was by preventing lipid peroxidation, measured by serum malondialdehyde (MDA) level [182].

Nyam et al. [190] applied an animal inflammatory model to illustrate kenaf seed extract’s capability to overcome histamine-, carrageenan-, and arachidonic acid-induced inflammation in rat paws. Results showed that KSE at dose 500 mg/kg body weight significantly reduced the size of edema in the rat paws, possibly by blocking cyclooxygenase (COX), lipoxygenase (LOX), and histamine activities that have crucial roles in inflammation [190].

Moreover, kenaf seed also portrays unusual antimicrobial activity against bacteria such as *Bacillus cereus*, *Escherichia coli*, and *Bacillus subtilis* by effectively producing a large zone of inhibition in a disc diffusion assay [181].

## 5. Conclusions

The current review has revealed the beneficial health effects of plant-based foods from the seeds of several fibre crops contributed by their various nutritive values and bioactive phenolic compounds. Kenaf, hemp, cotton, and okra are several important fibre crops widely exploited for their fibrous stems to meet increasing human demands for economic reasons. These underutilized seeds are highly valuable food sources and ingredients, with many of them having been consumed in some countries for a long time in bakery products and beverages. A wide array of polyphenolic compounds that possess biological activities (i.e., anti-inflammatory, antidiabetic, anticancer, antineurodegenerative, anti-anxiety, and antimicrobial) are contained in these seeds, as proven in many scientific investigations. Hence, effective utilization of these highly nutritious by-products can significantly be used as one of the means to curb poverty, enhance food security, and reduce environmental issues, in line with the United Nations’ Sustainable Development Goals (SDGs).

## Figures and Tables

**Table 1:** Major classes of polyphenols, selected polyphenolic compounds, and their beneficial health effects, obtained from underutilized seeds.

Polyphenolic Class	Polyphenolic Compound	Molecular Formulae	Beneficial Health Effects	Sources	References
Phenolic acids	Gallic acid	C_7_H_6_O_5_	Anti-inflammatory, antidiabetic, anti-obesity, antimicrobial, antineurodegenerative, anti-myocardial ischemia, hepaprotective	Grape seed, raspberry seed, flaxseed, date seed, corn seed, lime seed, guava seed	[43,44,45,46,47,48]
	Syringic acid	C_9_H_10_O_5_	Antibacterial, hepatoprotective	Date seed, grape seed	
	Vanillic acid	C_8_H_8_O_4_	Anti-ulcer, anthelmintic, hepaprotective, neuroprotective, wound healing	Date seed, pumpkin seed, papaya seed, orange seed, grape seed	
	Chlorogenic acid	C_16_H_18_O_9_	Anti-obesity, antidiabetic, antimicrobial, anticarcinogenic	Apple seed, sunflower seed, chia seed, coffee bean, date seed, lime seed, orange seed	
	Caffeic acid	C_9_H_8_O_4_	Anti-inflammatory, anticarcinogenic, antidiabetic, antineurodegenerative	Chia seed, date seed, lime seed, orange seed, guava seed	
	Ferulic acid	C_10_H_10_O_4_	Anti-ageing, antidiabetic, antimicrobial	Oat seed, corn seed, date seed, lime seed, orange seed, grape seed	
	p-hydroxybenzoic acid	C_7_H_6_O_3_	Antimicrobial, antimutagenic	Papaya seed, grape seed	
	p-coumaric acid	C_9_H_8_O_3_	Anti-inflammatory, antineoplastic, antimicrobial, anti-platelet aggregation, antidiabetic, neuroprotective	Grape seed, lime seed, orange seed	
	Protocatechuic acid	C_7_H_6_O_4_	Antibacterial, anticancer, anti-ulcer, anti-ageing, analgesic	Apple seed, berry seed, date seed, lime seed, orange seed, grape seed	
	Caffeoylquinic acid	C_16_H_18_O_9_	Anti-inflammatory, antidiabetic	Date seed	
	Caffeoylshikimic acid	C_16_H_16_O_8_	Anticancer	Date seed	
Flavonoids	Catechin	C_15_H_14_O_6_	Anti-allergic, anti-ageing, anticancer, antimicrobial	Avocado seed, lime seed	[47,48,49,50,51]
	Quercetin	C_15_H_10_O_7_	Anti-arthritic, anti-inflammatory, antihypertensive, anticancer, antineurodegenerative, cardioprotective, wound healing	Quinoa seed, chia seed, lime seed, grape seed	
	Cyanidin	C_15_H_11_O_6_+	Anti-inflammatory, antidiabetic	Black soybean, purple corn seed, mulberry seed, guava seed	
	Kaempferol	C_15_H_10_O_6_	Anticancer, antimicrobial, cardioprotective, neuroprotective	Red bean, pinto bean, quinoa seed, lime seed	
	Rutin	C_27_H_30_O_16_	Anti-allergic, antiproliferative	Tomato seed, orange seed	
	Apigenin	C_15_H_10_O_5_	Antidiabetic	Celery seed	
	Luteolin	C_15_H_10_O_6_	Anti-inflammatory, antidiabetic	Celery seed	
	Naringenin	C_15_H_12_O_5_	Anticancer, antidiabetic, antimutagenic	Celery seed, grapefruit seed, tomato seed, lime seed, orange seed	
	Hyperin	C_21_H_2_0O_12_	Antihyperglycemic, antiviral, anti-ulcer, antinociceptive, anticancer, hepatoprotective, myocardial protection	Apple seed	
	Phloridzin	C_21_H_24_O_10_	Antidiabetic, antimicrobial	Apple seed, pumpkin seed	
Lignans	Arctigenin	C_21_H_24_O_6_	Anti-inflammatory, anticancer, antimicrobial, antiviral	Greater burdock seed	[52,53,54,55]
	Secoisolariciresinol	C_20_H_26_O_6_	Anticancer, anti-estrogenic, cardioprotective	Flaxseed, sunflower seed, pumpkin seed, sesame seed	
	Matairesinol	C_20_H_22_O_6_	Anti-inflammatory	Flaxseed, sesame seed, grape seed	
	Sesamin	C_20_H_18_O_6_	Anti-inflammatory, anti-ageing, anti-estrogenic, anticancer, antimicrobial, neuroprotective	Sesame seed, cashew nut	
	Sesaminol	C_20_H_18_O_7_			
	Sesamol	C_7_H_6_O_3_			
	Sesamolinol	C_20_H_20_O_7_			
Stillbenes	Resveratrol	C_14_H_12_O_3_	Antihyperglycemic, antihypercholesterolemic, anticancer, anti-obesity, antimutagenic	Grape seed, passion fruit seed	[56,57,58]
	Pterostilbene	C_16_H_16_O_3_	Anticancer, cardioprotective, antimicrobial	Grape seed	
	Piceatannol	C_14_H_12_O_4_	Anti-obesity, antihyperglycemic, anticancer, skin protective	Passion fruit seed	
	Pinosylvin	C_14_H_12_O_2_	Anti-inflammatory, antimicrobial, anticancer	Grape seed	

## Data Availability

Not applicable.
